# Complete fatty degeneration of thymus associates with male sex, obesity and loss of circulating naïve CD8^+^ T cells in a Swedish middle-aged population

**DOI:** 10.1186/s12979-023-00371-7

**Published:** 2023-08-31

**Authors:** Mårten Sandstedt, Rosanna W S Chung, Camilla Skoglund, Anna K. Lundberg, Carl Johan Östgren, Jan Ernerudh, Lena Jonasson

**Affiliations:** 1 Center for Medical Image Science and Visualization (CMIV), Linköping University, Linköping, Sweden; 2 Department of Radiology and Department of Health, Medicine and Caring Sciences, Faculty of Medicine and Health Sciences, Linköping University, Linköping, Sweden; 3 Department of Health, Medicine and Caring Sciences, Faculty of Medicine and Health Sciences, Linköping University, Linköping, Sweden; 4 Department of Clinical Immunology and Transfusion Medicine and Department of Biomedical and Clinical Sciences, Faculty of Medicine and Health Sciences, Linköping University, Linköping, Sweden; 5 Department of Cardiology and Department of Health, Medicine and Caring Sciences, Faculty of Medicine and Health Sciences, Linköping University, Linköping, Sweden

**Keywords:** Thymus, T cell, Inflammation, Aging, Sex, Obesity

## Abstract

**Background:**

Fatty degeneration of thymus (or thymus involution) has long been considered a normal ageing process. However, there is emerging evidence that thymic involution is linked to T cell aging, chronic inflammation and increased morbidity. Other factors, aside from chronological age, have been proposed to affect the involution rate. In the present study, we investigated the imaging characteristics of thymus on computed tomography (CT) in a Swedish middle-aged population. The major aims were to establish the prevalence of fatty degeneration of thymus and to determine its associations with demographic, lifestyle and clinical factors, as well as inflammation, T cell differentiation and thymic output.

**Results:**

In total, 1 048 randomly invited individuals (aged 50–64 years, 49% females) were included and thoroughly characterized. CT evaluation of thymus included measurements of attenuation, size and a 4-point scoring system, with scale 0–3 based on the ratio of fat and soft tissue. A majority, 615 (59%) showed complete fatty degeneration, 259 (25%) predominantly fatty attenuation, 105 (10%) half fatty and half soft-tissue attenuation, while 69 (6.6%) presented with a solid thymic gland with predominantly soft-tissue attenuation. Age, male sex, high BMI, abdominal obesity and low dietary intake of fiber were independently associated with complete fatty degeneration of thymus. Also, fatty degeneration of thymus as well as low CT attenuation values were independently related to lower proportion of naïve CD8^+^ T cells, which in turn was related to lower thymic output, assessed by T-cell receptor excision circle (TREC) levels.

**Conclusion:**

Among Swedish middle-aged subjects, nearly two-thirds showed complete fatty degeneration of thymus on CT. This was linked to depletion of naïve CD8^+^ T cells indicating that CT scans of thymus might be used to estimate immunological aging. Furthermore, our findings support the intriguing concept that obesity as well as low fiber intake contribute to immunological aging, thereby raising the possibility of preventive strategies.

**Supplementary Information:**

The online version contains supplementary material available at 10.1186/s12979-023-00371-7.

## Background

T cells have a fundamental role in the establishment of adaptive immune response. They maintain a lifelong immune homeostasis but can also act as major drivers of inflammatory and autoimmune diseases. Thymus is the primary lymphoid organ being responsible for the generation of T cells. During infancy, T cell emigrants from thymus establish the circulating pool of naïve CD4^+^ T cells, including naive CD4^+^ regulatory T (T_reg_) cells, and naïve CD8^+^ T cells [1]. After puberty, thymic output and function progressively decreases with age due to a process termed thymic involution. Anatomically, thymus gradually atrophies, its perivascular space expands and fills with adipose tissue. However, the rate of thymic involution may vary among individuals. Recent data, mainly based on experimental research, reveals that not only age, but also other factors influence thymic involution such as sex hormones, obesity, autoimmune diseases, infections and oxidative stress [2, 3]. Still, it remains unclear whether thymic involution plays any significant role in adults. A commonly held belief has been that thymus is not important in adult life since homeostatic proliferation of peripheral naïve T cells compensates for the diminishing thymic output. On the other hand, an increasing body of evidence indicates that thymic involution is a primary hallmark of T cell aging, which is accompanied by immunosenescence and inflammaging. Immunosenescence is the gradual decline in immune function that occurs with age, while inflammaging is referred to as the chronic low-grade systemic inflammation observed in elderly in the absence of overt infection [4, 5]. Both immunosenescence and inflammaging are associated with the emergence of age-related diseases, such as dementia and cardiovascular diseases. Furthermore, a strong link between thymic involution and incidence of both infectious diseases and cancer has been proposed [2]. In accordance, early thymectomy in infants has demonstrated a subsequent premature aging of the immune system [6, 7] and increased incidence of infections, cancer and autoimmunity at follow-up after 18 years [8]. Moreover, surrogate markers of thymic function have been shown to predict allogeneic hematopoietic cell transplantation outcome in adults [9, 10] as well as survival in healthy elderly [11]. Interestingly, strategies aiming to regenerate thymus or boost thymic function are currently investigated [12].

The development of computerized tomography (CT) scanner technology has significantly improved the imaging evaluation of thymus. Thereby, it has become evident that residual thymic tissue in adults is more prevalent than previously thought [13, 14]. Also, emerging evidence indicates that visual assessment of CT scans with thymic scores based on relative proportions of fat and soft tissue may create a useful index of thymic involution [13–15]. Araki et al. [13] investigated the CT appearance of thymus in a cohort of 2 540 participants aged 34–92 years, and found a high prevalence of fatty acid degeneration and soft tissue attenuation in thymus. They further reported that age, male sex, former smoking and high body mass index (BMI) were independently associated with increased fatty degeneration of thymus. This is so far the only study that has investigated the CT appearance of thymus and its associations in a large human sample. Ackman et al. [15] used the same scoring system to assess sex differences in thymus appearance in a minor sample of young adults and found a significantly higher fat content in male thymuses.

It is still unclear whether visualization of thymus on CT is appropriate to draw any conclusions on thymus function, in terms of thymic output or size of the naïve T cell pool. Thymic output can be estimated by circulating numbers of naïve T cells and recent thymic emigrants, the latter defined by the presence of T cell receptor excision circles (TRECs), representing rest products from the intrathymic T cell receptor rearrangement in recently emigrated T cells [16]. So far, a few studies have proposed that detectable thymic tissue on CT correlates with naïve T cell counts and TREC levels after autologous transplant in adults [17] and in human immunodeficiency virus type 1-infected patients [18, 19]. However, T cell status in relation to CT thymic appearance has not been investigated at a general population level. Increased knowledge about thymus and factors that are associated with its function and role in inflammation could lead to new strategies and potential treatments in a broad range of health conditions.

In the present study, we investigated the CT appearance of thymus in a Swedish general population aged 50–64 years. The participants were thoroughly characterized regarding lifestyle and clinical factors, as well as inflammation, T cell differentiation and thymic output. The major aims were to establish whether the prevalence and degree of fatty degeneration of thymus was associated with demographic, lifestyle and clinical factors, and if it had any impact on low-grade systemic inflammation and circulating naïve subsets of CD4^+^ and CD8^+^ T cells.

## Methods

### Study population

The Swedish cardiopulmonary bio-image study (SCAPIS) is a general population-based prospective study [20] conducted at six Swedish university hospitals in Gothenburg, Linköping, Malmö/Lund, Stockholm, Umeå and Uppsala, with each site recruiting participants from corresponding municipality areas. Between 2013 and 2018, over 30 000 participants aged 50–64 years were invited to a comprehensive examination. Over a period of two days, the participants underwent extensive imaging and functional studies of the heart, lungs and metabolism and completed extensive questionnaires including lifestyle and medication. The SCAPIS Leukocyte substudy was undertaken to assess the immune profile by flow cytometry in fresh whole blood samples. Between November 2015 and May 2018, 1 077 participants in SCAPIS Leukocyte were consecutively recruited from the SCAPIS Linköping cohort. We strived for gender balance but otherwise no exclusion criteria were applied except the inability to understand written and spoken Swedish for informed consent.

### Flow cytometry analysis of T cell subsets

To obtain absolute counts of CD3 + , CD4^+^ and CD8^+^ T cells, EDTA-whole blood was stained with anti-CD3 FITC, anti-CD8 PE, anti-CD45 PerCP and anti-CD4 APC antibodies using BD Multitest Trucount tubes (Becton, Dickinson and Company, Franklin Lakes, US) according to the manufacturer’s instructions. Proportions of naïve T cells including naïve T_reg_ cells within the CD4^+^ compartment, as well as naïve T cells within the CD8^+^ T cell compartment were determined by multi-colour whole blood staining using the following monoclonal antibodies: anti-CD3 PerCP (Clone:SK7), anti-CD4 PE-Cy7 (Clone: SK3), anti-CD8 APC-H7 (Clone: SK1), anti-CD45RA FITC (Clone: L48) and anti-CCR7 BV421 (Clone: 150,503). Four µL of each antibody were used to stain 50 µL EDTA-whole blood for 15 min in room temperature in the dark. Erythrocytes were lysed using 1 × FACS Lysing Solution for 15 min in room temperature. After incubation, cell supernatant was separated by two rounds of centrifugation (300 × g, 20ºC for 5 min). 2 mL and 500 µL of phosphate-buffered saline / 0.4% (w/v) human serum albumin solution was mixed with the supernatant after the first and second round of centrifugation, respectively. The cells were then analyzed on a FACS Canto II flow cytometer by using FACSDiva software (BD Biosciences). All reagents were purchased from BD Biosciences, Sweden. Naïve CD4^+^ and CD8^+^ T cell subsets were defined as CD45RA^+^CCR7^+^ cells within the CD3^+^CD4^+^ and CD3^+^CD8^+^ populations, respectively. Naïve T_reg_ cells were defined as CD25^++^CD45RA^+^ within the CD3^+^CD4^+^ population, as described by Hellberg et al. [21]. The gating strategy for CD3^+^, CD4^+^ and CD8^+^ T cells is shown in Supplementary Fig. 1, and the gating strategy for naïve CD4 + T cells, naive CD8 + T cells and naïve T_reg_ cells in Supplementary Fig. 2.

### Cytokine measurements

IL-6 and IL-18 in EDTA plasma samples were analyzed at SciLifeLab Affinity Proteomics Uppsala, Sweden, using the U-Plex Meso Scale Discovery (MSD) platform (Rockville, Maryland, USA), an electrochemiluminescence assay, according to the manufacturer’s instructions. Inter-assay coefficients of variations (CV) were 21.3% for IL-6 and 10,6% for IL-18.

### Droplet digital PCR for TRECs

In a subset of 55 consecutively recruited individuals, peripheral blood mononuclear cells were isolated using Ficoll gradient centrifugation and stored until use in liquid nitrogen. DNA purification was performed using QIAamp DNA Mini Kit (Protocol: DNA purification from blood or body fluids—spin protocol, Qiagen), according to the manufacturer’s instructions. Levels of TRECs were analyzed with droplet digital PCR (BioRad). For the amplification of TRECs a custom assay from BioRad was used containing the following sequences: forward primer 5’-CAC ATC CCT TTC AAC CAT GCT-3’ (900 nM), reverse primer 5’-GCC AGC TGC AGG GTT TAG G-3’ (900 nM), probe 5’-ACA CCT CTG TTT TTG TAA AGG TGC CCA CT-3’ (dye 5′6-FAM, quencher 3’ Iowa Black FQ, 250 nM). A reaction mix consisting of supermix (ddPCR SMX for Probes, no dUTP, cat no 1863023, BioRad), TREC assay (described above), distilled water and Hind III reaction enzyme (5 U/reaction, recombinant 10.000 units, cat no R0104S, BioNordika) was added to DNA (1.1 µg DNA per reaction). After incubation for 20 min, droplets were generated using an automatic droplet generator (QX200 Droplet Generator, BioRad) followed by PCR amplification (C1000 Touch Thermal Cycler, BioRad). After incubation at + 4℃ overnight, the plate was inserted into a droplet reader (QX200 Droplet Reader, BioRad) and the results were presented as copies/µL in the Quantasoft Software (version 1.7.4, BioRad). A positive control (a 29-year-old healthy volunteer) and a no template control was included in each run.

### Imaging of thymus

In total, 1 077 participants were included. Of these, 29 (2.7%) were excluded; nine (0.8%) had missing images, six (0.6%) had anterior mediastinal lymphadenopathy/tumor, five (0.5%) had previous surgery preventing evaluation, two (0.2%) had artefacts and seven (0.6%) could not be evaluated due to miscellaneous anatomical and technical reasons. Thus, data from in total 1 048 participants were included in the final analysis.

According to the SCAPIS protocol [20], a CT of thorax was performed using a dedicated dual-source CT scanner equipped with a Stellar Detector (Somatom Definition Flash, Siemens Medical Solution, Forchheim, Germany). All scans were acquired with a low radiation dose protocol at maximum inspiration without intravenous contrast. Image acquisition parameters were as follows: tube voltage 120 kV, automatic current modulation (CARE Dose4D; Siemens Medical Solutions) quality ref 25 mAs, pitch 0.9 and rotation time 0,5 s. Detector configuration was 128 × 0,6 mm. Reconstructions used were performed with an iterative reconstruction soft tissue kernel (SAFIRE, I31), matrix size 512 × 512, slice thickness 3 mm, increment 2 mm.

**Fig. 1:**
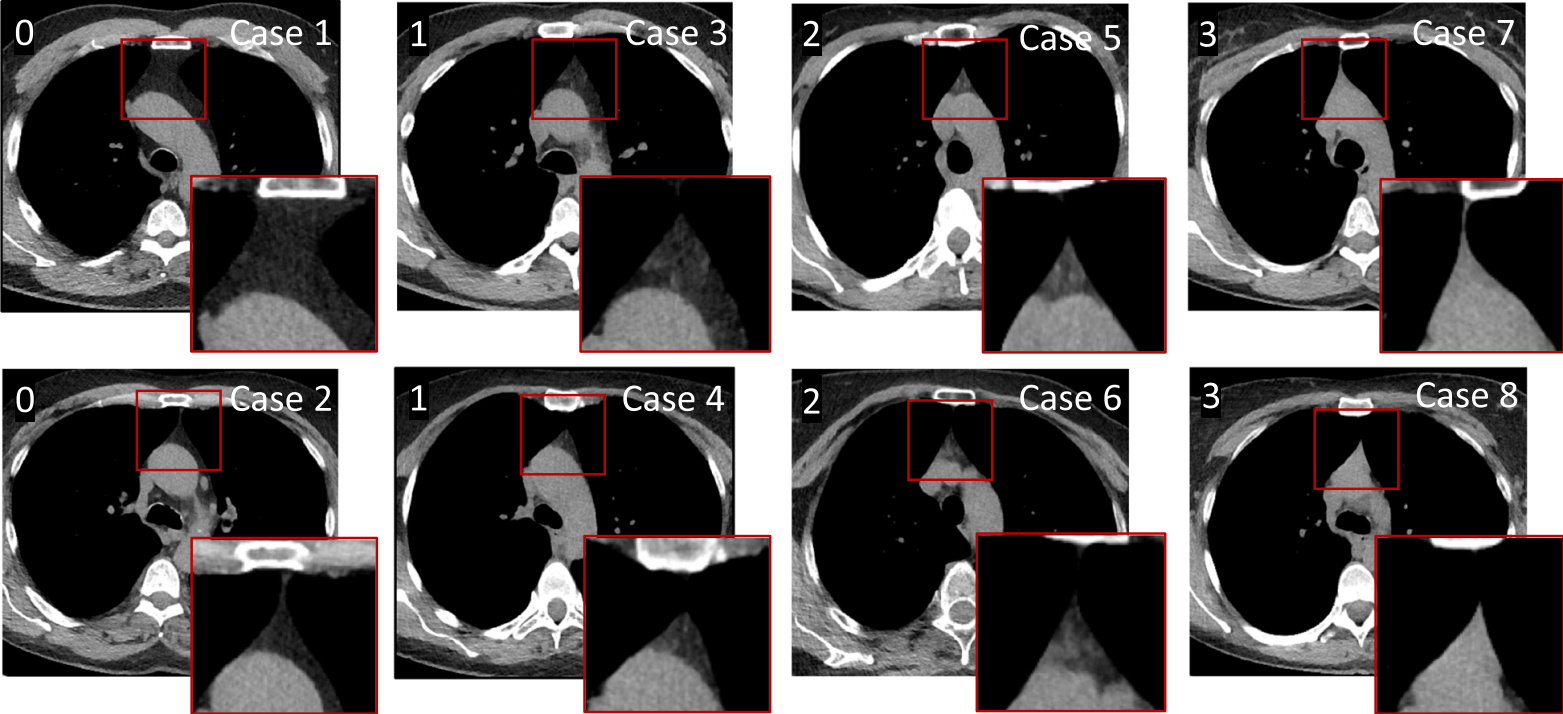
Representative CT images of each thymic score

All scans were reviewed using a fixed window setting (WL = 50, WW = 350) on a picture archiving and communication system station. Evaluations were performed by a thoracic radiologist with 19 years´ experience in interpreting chest CT scans. Thymus fat and soft tissue content was visually analyzed and graded on a scale 0–3, as previously described [13, 15]: grade 0, complete fatty replacement, no identifiable soft tissue in the thymic bed; grade 1, predominantly fatty thymus; grade 2, approximately one-half fatty and one-half soft tissue attenuation thymus; and grade 3, predominantly soft tissue attenuation thymus (Fig. 1).

Each thymic score (indicated at top left in the image) is represented by two different cases. Cases 1 and 2: Score 0, complete fatty replacement, no identifiable soft tissue in the thymic bed, Cases 3 and 4: Score 1, predominantly fatty thymus, Cases 5 and 6: Score 2, approximately one-half fatty and one-half soft tissue attenuation thymus, and Cases 7 and 8: Score 3, predominantly soft tissue attenuation thymus.

Quantitative measurements were performed in individuals with scores 1, 2 and 3, including thymic attenuation and thymic size (anteroposterior and transverse diameters, length and thickness of right and left lobes). An axial image with maximum anterior–posterior diameter of thymic gland was selected, and all following measurements and evaluations were performed in the same image [13, 22]. A region of interest for attenuation measurement in Hounsfield units (HU) was defined, encompassing thymic soft tissue, excluding as much adjacent fat tissue as possible [13, 22, 23]. The morphology was determined using the following categories: 1) pyramidal with convex margins, 2) pyramidal with straight margins, 3) pyramidal with concave margins, 4) round or oval, and 5) irregular [13]. If thymic lobes had different configurations, the dominant lobe was registered [13].

To assess intra- and inter-observer agreement of thymic scores, two thoracic radiologists reviewed 112 randomly selected CT scans. One radiologist scored the 112 cases twice and the second investigator scored the same 112 cases once. For those evaluations, the radiologists were blinded to SCAPIS data and previous radiological assessments.

### Covariate assessments

Weight was measured with participants in light clothing, using calibrated scales. BMI was calculated by dividing the weight (kg) by the square of the height (m). Abdominal obesity was defined as a waist circumference ≥ 102 cm in men and ≥ 88 cm in women. Office blood pressure was calculated as the average of three measurements, after 5-min supine rest, measured in the right brachial artery.

Sensor-based sedentary behavior and physical activity patterns were derived from tri-axial accelerometers (ActiGraph LCC, Pensacola, FL, USA), as previously described [24]. The participants wore the accelerometers for seven days. Wear time was calculated as 24 h minus non-wear time. Participants with a minimum of 600 min of valid daily wear time for at least 4 days were included. Total physical activity was expressed in daily mean tri-axial vector magnitude counts per minute (cpm). Sedentary time was defined as < 200 cpm, low intensity physical activity as 200–2689 cpm, moderate intensity physical activity as 2690–6166 cpm, and vigorous physical activity as ≥ 6167 cpm.

A shorter form of the food-frequency questionnaire Meal-Q (MiniMeal-Q) was used to assess dietary intake [25, 26]. The daily intake of energy, macronutrients, fiber and micronutrients was retrieved by linking intake of food items and dishes assessed with MiniMeal-Q to the Swedish food composition database provided by the Swedish National Food Agency and calculated as the average intake of units per day.

Plasma levels of high-sensitivity C-reactive protein (CRP) were measured using an immunoturbidimetric assay with a Roche Cobas C502 analyzer (Roche Diagnostics, Scandinavia AB), with a CV of 2.2%. Total cholesterol, high-density lipoprotein (HDL) cholesterol, low-density lipoprotein (LDL) cholesterol, triglycerides and glycated hemoglobin (HbA1c) were measured in a fasting condition at study entry through standard analyses at an accredited laboratory. White blood cell differential counts in whole blood were determined by Cell-Dyn Sapphire™ (Abbot Diagnostics).

### Statistical analyses

IBM SPSS Statistics (version 28) and GraphPad Prism (version 9.1.2) were used for analyses. Cohen´s weighed kappa coefficients were used to determine intra- and inter-observer agreements of thymic scores. The Kruskal–Wallis H test, alone or in combination with Dunn’s multiple comparisons test, or the Mann–Whitney test were used to compare characteristics between groups with different thymic scores. Spearman´s correlation was used to analyze univariate correlations between quantitative variables. Logistic regression was used to analyze which factors were associated with discrepancies between different thymic scores. Linear regression was used to determine independent determinants of CT attenuation and thymic size.

### Availability of data and material

The data that support the findings of this study are available from the corresponding author upon reasonable request.

## Results

### Thymic scores and quantitative measurements of thymus on CT

**Fig. 2:**
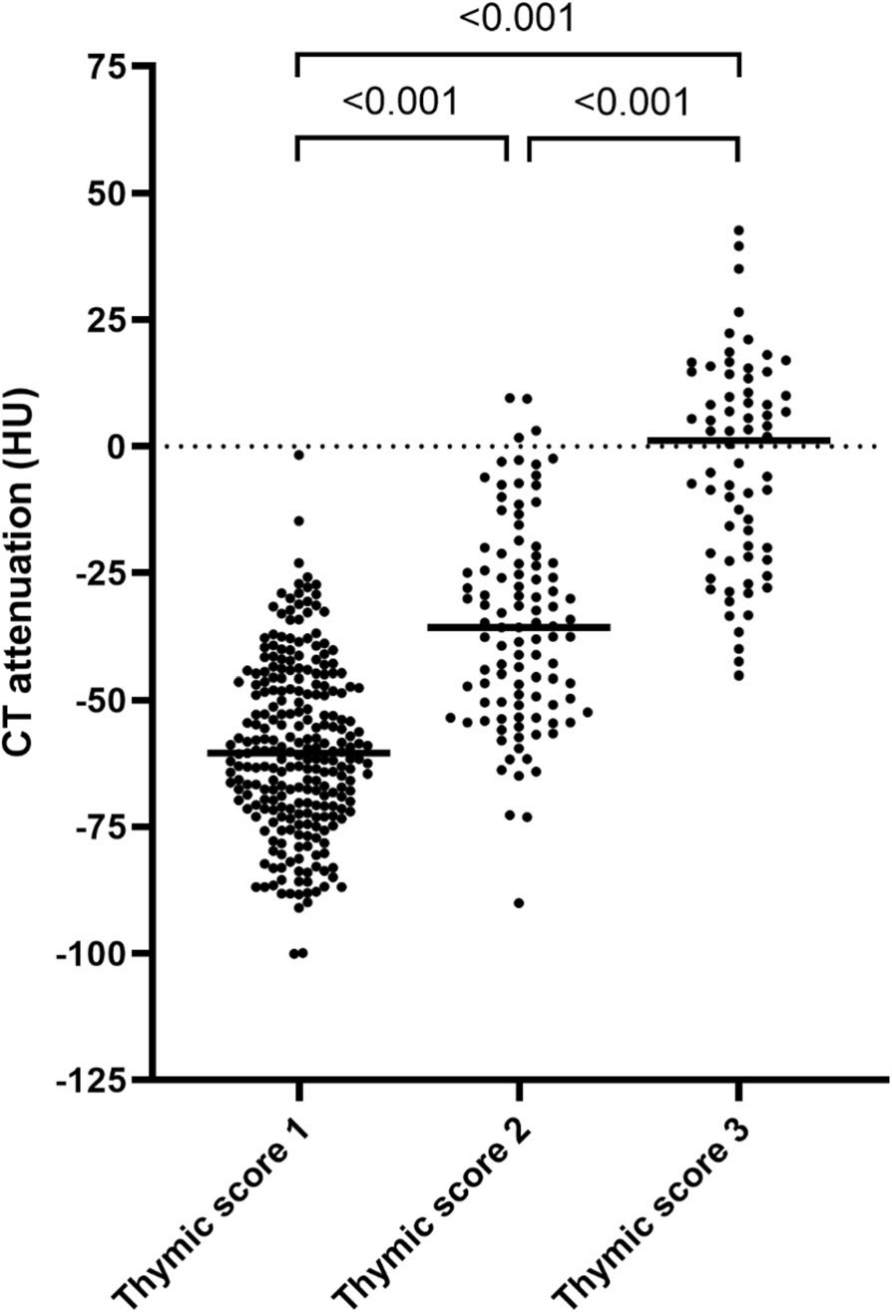
CT attenuation of thymic glands based on thymic scores

The distribution of thymic scores in the whole cohort was as follows: 615 (59%) had complete fatty degeneration of thymus (Score 0), 259 (25%) had predominantly fatty attenuation (Score 1), 105 (10%) had half fatty and half soft-tissue attenuation (Score 2), and 69 (6.6%) had a solid thymic gland with predominantly soft-tissue attenuation (Score 3). The CT attenuation values of the thymic gland were determined in participants with Scores ≥ 1. As shown in Fig. 2, the attenuation values progressively decreased from Score 3 to Score 1 (*p* < 0.001), thus confirming the presence of less dense tissue in those with lower scores. All thymic size measurements (anteroposterior and transverse diameters, length and thickness of right and left lobes) showed significantly higher values in subjects with Score 1 compared to those with higher thymic scores (Supplementary Table 1). Morphologically, the most common shape of thymus was pyramidal with straight margins, 294 (70%), followed by pyramidal with convex margins, 69 (17%), pyramidal with concave margins, 34 (8%) and irregular, 22 (5%). Intra- and inter-observer agreements of thymic scores were good, showing weighed Cohen kappa coefficients of 0.72 (95% CI: 0.59–0.84) and 0.68 (95% CI: 0.54–0.79), respectively. Details of agreements are shown in Supplementary Table 2a-b.

CT attenuation values are expressed as Hounsfield units (HU), in subjects with thymic scores ≥ 1; Score 1, predominantly fatty thymus, Score 2, approximately one-half fatty and one-half soft tissue attenuation thymus, and Score 3, predominantly soft tissue attenuation thymus. Kruskal–Wallis H test followed by Dunn’s multiple comparisons test was used for the analysis.

### Complete fatty degeneration of thymus associates with age, male sex, obesity, hypertension, diabetes, dyslipidemia and inflammation

Demographic, clinical and laboratory characteristics of participants divided into four groups based on their thymic scores are displayed in Table 1. The majority (64%) of subjects with Score 0 were male while the vast majority of those with higher scores were female, 75% with Score 2 and 85% with Score 3. Higher BMI values, as well as abdominal obesity, were more frequent in subjects with lower scores. Those with Score 0 were also more likely to be former smokers. The use of anti-hypertensive and anti-diabetic medication did not differ across groups. However, hypertension defined as systolic blood pressure ≥ 140 mm Hg or diastolic blood pressure ≥ 90 Hg was more common in those with Score 0 compared to those with higher scores, and so was HbA1c ≥ 48 mmol/mol, indicating diabetes. Also, dyslipidemia, defined as lower levels of HDL cholesterol and higher levels of triglycerides, was a more common finding in subjects with Score 0. Furthermore, higher leukocyte and granulocyte counts and higher levels of CRP, IL-6 and IL-18 revealed the presence of a proinflammatory state in those with Score 0. Given the skewed distribution of male and female participants across thymic scores, the demographic, clinical and laboratory characteristics are shown separately for male and female participants in Supplementary Table 5 and 6, respectively.

**Table 1:** Characteristics of participants based on thymic scores. Four groups of thymic scores are presented; Score 0, complete fatty replacement, no identifiable soft tissue in the thymic bed, Score 1, predominantly fatty thymus, Score 2, approximately one-half fatty and one-half soft tissue attenuation thymus, and Score 3, predominantly soft tissue attenuation thymus

	**Thymic scores**				
	0 (*n* = 615)	1 (*n* = 259)	2 (*n* = 105)	3 (*n* = 69)	p^a^
*Demographic and clinical characteristics*					
Age, years	58 (55–62)	56 (52–60)	54 (52–59)	54 (52–59)	< 0.001
Male sex	391 (64)	103 (40)	26 (25)	10 (14)	< 0.001
BMI	27 (25–30)	25 (24–28)	24 (23–27)	24 (21–26)	< 0.001
Abdominal obesity	488 (79)	144 (56)	40 (38)	16 (23)	< 0.001
Smoking, current former	52 (8.5)	12 (4.6)	6 (5.7)	4 (5.8)	NS
	204 (33)	71 (27)	26 (25)	11 (16)	0.009
Anti-hypertensive medication	136 (22)	38 (15)	3 (2.9)	8 (12)	NS
Hypertension, systolic^b^ diastolic^c^	209 (34)	70 (27)	20 (19)	16 (23)	0.003
	168 (27)	52 (20)	16 (15)	13 (19)	0.009
Anti-diabetic medication	40 (6.5)	4 (1.5)	3 (2.9)	2 (2.9)	NS
Diabetes^d^	61 (9.9)	4 (1.5)	3 (2.9)	5 (7.2)	0.009
*Clinical chemistry*					
Total cholesterol, mmol/L	5.4 (4.6–6.2)	5.4 (4.8–6.1)	5.4 (4.8–6.3)	5.2 (4.8–5.9)	NS
LDL cholesterol, mmol/L	3.2 (2.6–4.0)	3.2 (2.8–3.8)	3.2 (2.5–3.9)	2.8 (2.3–3.3)	0.035
HDL cholesterol, mmol/L	1.5 (1.2–1.8)	1.7 (1.4–2.0)	1.7 (1.5–2.2)	2.0 (1.8–2.4)	< 0.001
Triglycerides, mmol/L	1.2 (0.8–1.6)	1.0 (0.7–1.3)	0.9 (0.7–1.2)	0.8 (0.6–1.0)	< 0.001
CRP, mg/L	1.1 (0.6–2.3)	0.9 (0.4–1.7)	0.7 (0.3–1.4)	0.8 (0.3–1.5)	< 0.001
IL-6, pg/mL	1.15 (0.80–1.57)	1.02 (0.68–1.47)	0.96 (0.68–1.39)	1.02 (0.77–1.43)	0.004
IL-18, pg/mL	365 (282–464)	323 (251–406)	310 (244–432)	272 (222–369)	< 0.001
*White blood cell differential counts*					
Leukocytes, 10^3^/μL	5.9 (5.1–7.0)	5.5 (4.7–6.5)	5.3 (4.4–6.5)	5.4 (4.4–6.4)	< 0.001
Granulocytes, 10^3^/μL	3.5 (2.8–4.2)	3.1 (2.6–3.7)	2.9 (2.3–3.8)	2.8 (2.3–3.8)	< 0.001
Lymphocytes, 10^3^/μL	1.8 (1.5–2.1)	1.8 (1.5–2.1)	1.9 (1.5–2.2)	1.7 (1.5–2.1)	NS

Continuous data are expressed as median (interquartile range) and dichotomous data as n (%)

^a^The Kruskal–Wallis H test was used to compare characteristics between groups with different thymic scores

^b^Systolic hypertension, defined as ≥ 140 mm Hg

^c^Diastolic hypertension, defined as ≥ 90 mm Hg

^d^Diabetes, defined as HbA1c ≥ 48 mmol/mol

### Complete fatty degeneration of thymus associates with sedentary behavior and low intake of fibers and micronutrients

Data on physical activity and dietary intake are presented in Table 2. Subjects with lower thymic scores were found to spend higher time in sedentary and less time in all physical activity intensities compared with those with higher scores. As regards dietary intake, there were no differences in energy and macronutrient intake (carbohydrates, protein, total fat) across groups. However, the intake of fiber and micronutrients was significantly lower in those with complete fatty degeneration of thymus. Micronutrients that showed significant differences across groups at *p*-values < 0.01 are included in Table 2. A complete list of micronutrients based on thymic scores is given in Supplementary Table 3. Correlations between micronutrients are shown in Supplementary Table 4. Data on physical activity and dietary intake are shown separately for male and female participants in Supplementary Table 5 and 6, respectively.

**Table 2:** Physical activity levels and dietary intake in participants based on thymic scores. Physical activity is based on accelerometer-derived data, and calculated nutrients/day of energy, macronutrients, fiber and micronutrients are based on the food frequency questionnaire MiniMeal-Q. Four groups of thymic scores are presented; Score 0, complete fatty replacement, no identifiable soft tissue in the thymic bed, Score 1, predominantly fatty thymus, Score 2, approximately one-half fatty and one-half soft tissue attenuation thymus, and Score 3, predominantly soft tissue attenuation thymus

	**Thymic scores**				*p*^a^
	0 (*n* = 612)	1 (*n* = 259)	2 (*n* = 105)	3 (*n* = 69)	
**Physical activity,** % of daily wear time					
Sedentary	56 (49–63)	54 (47–60)	53 (46–58)	52 (46–57)	< 0.001
Low intensity	37 (32–45)	39 (34–46)	39 (35–46)	41 (36–47)	0.003
Moderate-vigorous	5.0 (4.0–7.0)	6.0 (4.0–8.0)	7.0 (5.0–8.0)	7.0 (5.0–8.5)	< 0.001
	0 (*n* = 615)	1 (*n* = 258)	2 (*n* = 103)	3 (*n* = 67)	
**Calculated nutrients**					
Energy (kcal)	1548 (1230–2019)	1596 (1278–1957)	1690 (1325–1909)	1465 (1167–1920)	NS
Protein (g)	63 (51–79)	65 (50–79)	64 (54–77)	58 (50–76)	NS
Carbohydrates (g)	163 (125–219)	169 (135–219)	168 (135–205)	164 (120–216)	NS
Total fat (g)	62 (47–82)	62 (48–82)	68 (51–81)	57 (45–79)	NS
Fiber (g)	16 (12–22)	19 (13–25)	20 (14–26)	20 (13–25)	0.000
β-carotene (μg)	2837 (1669–4829)	3502 (1959–5505)	3691 (2227–6260)	3768 (1988–6625)	0.000
Vitamin E (mg)	8.2 (6.1–11)	8.7 (6.6–12)	9.4 (7.2–12)	9.1 (6.1–11)	0.006
Vitamin K (μg)	24 (17–35)	27 (20–38)	28 (20–42)	28 (19–39)	0.000
Thiamine (mg)	1.1 (0.8–1.4)	1.2 (0.9–1.5)	1.2 (0.9–1.6)	1.2 (0.9–1.6)	0.003
Folate (μg)	276 (213–356)	307 (241–382)	319 (252–377)	307 (234–379)	0.000
Iron (mg)	8.9 (6.7–12)	10 (8.0–13)	10 (7.4–14)	9.7 (7.6–12)	0.004
Magnesium mg)	303 (245–384)	332 (270–418)	361 (273–420)	329 (249–422)	0.000

Data are expressed as median (interquartile range)

^a^The Kruskal–Wallis H test was used to compare characteristics between groups with different thymic scores

### Complete fatty degeneration of thymus associates with reduction of naïve T cell subsets

**Fig. 3:**
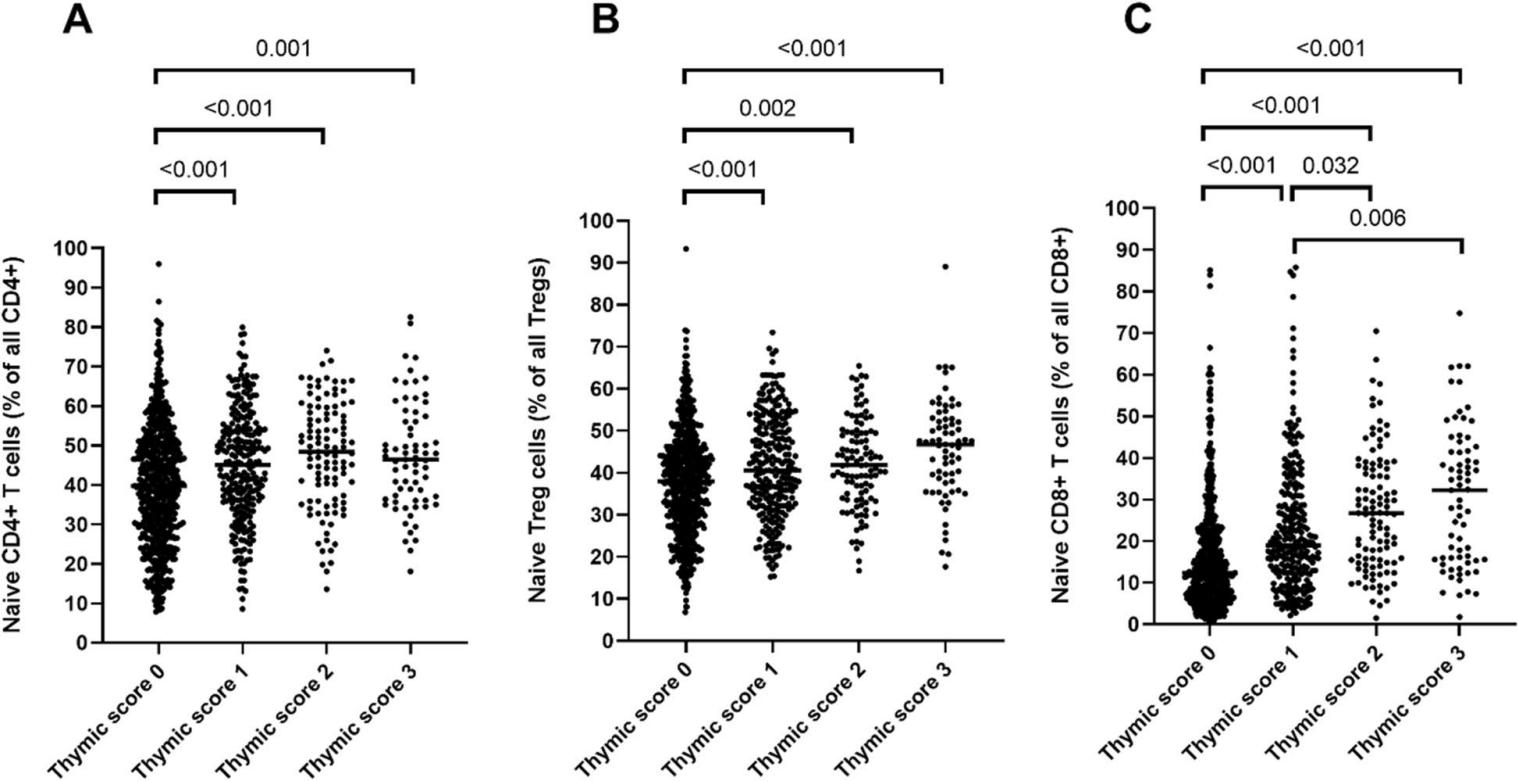
Proportions of naïve T cells within the CD4^+^ and CD8^+^ T cell compartments based on thymic scores Proportions of naïve subsets in naïve CD4^+^ T cells (**A**), naïve regulatory T (T_reg_) cells (**B**) and naïve CD8^+^ T cells (**C**). The Kruskal-Wallis H test showed *p* < 0.001 for all three analyses and was followed by Dunn’s multiple comparisons test

Regarding the major populations of T cells (total CD3^+^, CD4^+^ T cells, CD8^+^ T cells or T_reg_ cells), there were no differences across thymic score groups, neither in absolute numbers, nor in proportions (Table 3). In contrast, subset analysis showed that proportions of naïve cell (CD45RA^+^) were significantly reduced at lower thymic scores within the CD4^+^, CD8^+^ and T_reg_ populations (Fig. 3). In particular, the reduction of naïve CD8^+^ T cells within the CD8^+^ T cell population was pronounced at Score 0 showing almost threefold lower levels compared to Score 3, 12 (7.2–22) % *vs* 32 (16–42) %, *p* < 0.001. Data on major populations of T cells as well as naïve T cell subsets are shown separately for male and female participants in Supplementary Table 5 and 6, respectively.

**Table3:** T cells and T cell subsets based on thymic scores Flow cytometric data on CD3^+^ T cells and subsets of CD4^+^ T cells, regulatory T cells and CD8^+^ T cells in whole blood are presented based on thymic scores; Score 0, complete fatty replacement, no identifiable soft tissue in the thymic bed, Score 1, predominantly fatty thymus, Score 2, approximately one-half fatty and one-half soft tissue attenuation thymus, and Score 3, predominantly soft tissue attenuation thymus

	**Thymic scores**				
	**0 (*n* = 615)**	**1 (*n* = 259)**	**2 (*n* = 105)**	**3 (*n* = 69)**	p^a^
CD3^+^ T cells/μL	1317 (1076–1622)	1331 (1090–1630)	1432 (1131–1715)	1296 (1081–1665)	NS
CD4^+^ T cells/μL	852 (680–1070)	865 (674–1072)	882 (729–1166)	858 (670–1042)	NS
CD8^+^ T cells/μL	401 (287–584)	438 (314–582)	450 (332–598)	419 (376–564)	NS
CD4^+^ T cells, % of CD3^+^	67 (58–74)	67 (59–72)	66 (60–71)	65 (58–72)	NS
CD8^+^ T cells, % of CD3^+^	32 (25–41)	32 (26–39)	32 (27–39)	34 (27–41)	NS
T_reg_ cells, % of CD4^+^ cells	3.8 (3.2–4.6)	4.0 (3.3–4.8)	4.2 (3.4–4.9)	3.8 (3.3–4.7)	NS

T_reg_ cells, regulatory T cells. Data are expressed as median (interquartile range)

^a^The Kruskal-Wallis H test was used to compare characteristics between groups with different thymic scores

### Complete fatty degeneration of thymus associates with lower levels of TRECs

**Fig. 4:**
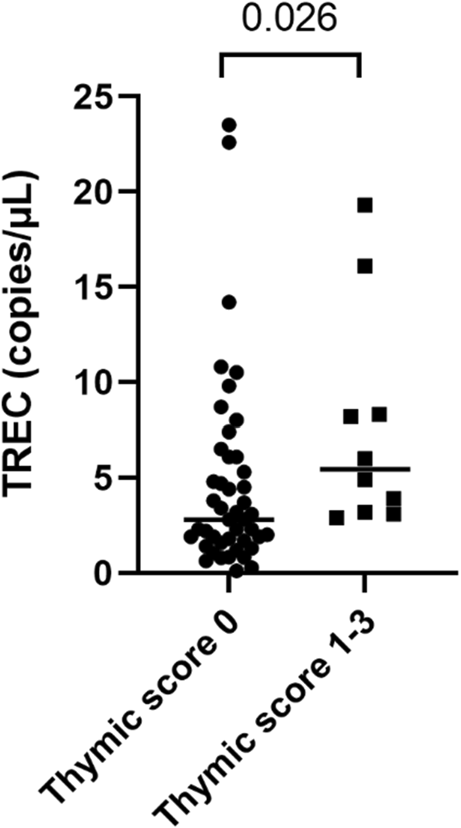
TRECs in peripheral blood mononuclear cells. T cell receptor excision circles (TRECs) were measured by droplet digital PCR in peripheral blood mononuclear cells from 55 individuals grouped by thymic score 0 (*n* = 45) and thymic score 1–3 (*n* = 10). The Mann–Whitney test was used for the analysis

TRECs in peripheral blood mononuclear cells were measured in a subsample of 55 individuals (Score 0, *n* = 45, Score 1–3, *n* = 10, mean age 59 years, 48 males). As shown in Fig. 4, significantly lower levels of TRECs were observed in subjects with Score 0 (median value 2.8 copies/µL) compared to those with Score 1–3 (median value 5.5 copies/µL), *p* = 0.026. Females (*n* = 7) had significantly higher levels of TRECs compared to males, *p* = 0.002. However, when females were excluded from the analysis, TREC levels were still lower in those with Score 0, *p* = 0.043. TRECs correlated significantly with the proportions of naïve CD8^+^ T cells (*r* = 0.405, *p* = 0.002) and naïve T_reg_ cells (*r* = 0.350, *p* = 0.009) but not with naïve CD4^+^ T cells (*r* = 0.139, *p* = 0.311).

### Age, male sex, obesity, dietary intake of fiber and proportions of naïve CD8^+^ T cells are independent determinant of complete fatty degeneration of thymus

Independent determinants of complete fatty degeneration of thymus (Score 0) were identified using logistic regression analyses (Table 4). Variables were included in the multiple logistic regression model if they had a *p* -value < 0.05 in univariate analysis, and if correlation coefficients between variables were < 0.60. The intake of several vitamins and minerals, except β-carotene, were strongly correlated (*r* > 0.60) with the intake of fiber, therefore fiber and β-carotene were included in the model. Thus, included variables were age, sex, BMI, abdominal obesity, hypertension, diabetes, smoking status, sedentary lifestyle, dietary intake of fiber and β-carotene, levels of HDL cholesterol, triglycerides, CRP, IL-6 and IL-18, granulocyte counts and proportions of naïve CD4^+^, naïve T_reg_ cells and naïve CD8^+^ T cells. When individuals with Score 0 (*n* = 615) were compared with those with Score 3 (*n* = 69), six variables including male sex, abdominal obesity, age, HDL cholesterol, naïve CD8^+^ T cells and naïve T_reg_ cells remained highly significant. When subjects with Score 0 were compared with all subjects with Scores ≥ 2 (*n* = 174) or with all subjects with Scores ≥ 1 (*n* = 433), male sex, abdominal obesity, age and naïve CD8^+^ T cells still remained independently associated with complete fatty degeneration of thymus. Furthermore, BMI and dietary intake of fiber entered as independent determinants in the last two models.

**Table 4:** Multiple logistic regression analysis of determinants of complete fatty degeneration of thymus. Independent determinants of complete fatty degeneration of thymus (Score 0) are presented. The group with Score 0 (*n* = 615) was compared with the following groups; Score 3, predominantly soft tissue (*n* = 69), Scores ≥ 2, at least one half fatty and one half soft-tissue attenuated thymus (*n* = 174), or Score ≥ 1, any detectable thymic tissue (*n* = 433)

	**Odds ratio**	**95% CI**	**p**
**Score 0** (*n* = 615) ***vs* Score 3** (*n* = 69)			
Age	1.14	1.05–1.24	0.002
Male sex	3.01	1.26–7.18	< 0.001
Abdominal obesity	3.33	1.58–7.01	< 0.001
HDL cholesterol	0.43	0.20–0.94	0.034
Naïve T_reg_ cells, % of all T_regs_	0.97	0.94–0.99	0.021
Naïve CD8^+^ T cells, % of all CD8^+^	0.95	0.93–0.97	< 0.001
**Score 0** (*n* = 615) ***vs* Score ≥ 2** (*n* = 174)			
Age	1.13	1.07–1.20	< 0.001
Male sex	3.38	1.95–5.84	< 0.001
Abdominal obesity	2.55	1.53–4.27	< 0.001
BMI	1.07	1.01–1.14	0.029
Naïve CD8 + T cells, % of all CD8^+^	0.96	0.95–0.97	< 0.001
Dietary intake of fiber	0.97	0.96–0.99	0.022
**Score 0** (*n* = 615) ***vs* Score ≥ 1** (*n* = 433)			
Age	1.10	1.06–1.14	< 0.001
Male sex	2.64	1.84–3.79	< 0.001
Abdominal obesity	1.79	1.25–2.56	0.001
BMI	1.09	1.04–1.14	< 0.001
Naïve CD8 + T cells, % of all CD8^+^	0.97	0.96–0.98	< 0.001
Dietary intake of fiber	0.98	0.97–0.99	0.027

Variables included but not retaining statistical significance in the models: Hypertension (systolic), diabetes, smoking status, sedentary behavior, dietary intake of b-carotene, granulocyte counts, levels of triglycerides, CRP, IL-6, IL-18, and proportions of naïve CD4^+^ T cells and naïve T_reg_ cells

Linear regression analysis was used to identify independent determinants of CT attenuation and thymic size in those with thymic scores ≥ 1 (Table 5). Variables were included in the linear regression analysis if they showed significant correlations (*p* < 0.05) with CT attenuation in univariate analysis. Independent determinants of CT attenuation were abdominal obesity, HDL cholesterol and proportions of naïve CD8^+^ T cells. The model explained 14% of the variation in attenuation. As regards thymic size (AP diameter), BMI, abdominal obesity, HDL cholesterol and male sex appeared to be independent determinants, explaining 28% of the variation in the model.

**Table 5:** Multiple linear regression analysis of determinants of CT attenuation and thymic size. Independent determinants of CT attenuation values are presented in subjects with any detectable thymic tissue (Score ≥ 1, *n* = 433) and thymic size in all subjects (*n* = 1078)

		β	**P**	**Adjusted R**^**2**^
**CT attenuation**^a^ (HU)	Abdominal obesity	-0.206	< 0.001	0.16
	HDL cholesterol	0.195	0.003	
	Naïve CD8 + T cells, % of all CD8^+^	0.165	0.002	
**Thymic size**^b^ (AP diameter, mm)	Abdominal obesity	0.150	0.006	0.28
	HDL cholesterol	-0.242	< 0.001	
	BMI	0.301	< 0.001	

*HU* Hounsfield units, *AP* Anteroposterior. β indicates standardized coefficient. Variables included but not retaining statistical significance in the models:

^a^age, sex, BMI, triglycerides, CRP

^b^age, triglycerides and CRP

## Discussion

The present study showed a large variation in CT appearance of thymus in a large population-based Swedish cohort aged 50–64 years. The availability of a broad arsenal of lifestyle and biological factors allowed us to define male sex, BMI, abdominal obesity and low dietary intake of fiber, in addition to age, as independently associated with fatty degeneration of thymus. Interestingly, fatty degeneration of thymus was also independently associated with lower proportion of naïve CD8^+^ T cells, which in turn was related to lower output of T cells from thymus, thus indicating a connection between thymic involution and immunological ageing.

Complete fatty replacement of thymus (Score 0) was present in 59% whereas thymic glands with half or more residual soft-tissue parenchyma (Scores 2 and 3) were seen in 16%. Despite a rather narrow age range of 50–64 years, the age showed independent association with Score 0. A couple of previous studies have used the similar scoring system to assess CT appearance of thymus in larger samples of middle-aged and elderly subjects. Araki et al. [13] investigated thymus in 2 540 subjects, aged 34–92 years, of the Framingham Heart Study cohort who underwent CT between 2008–2011. In the age groups 50–59 years (*n* = 771) and 60–69 years (*n* = 608), they found that Score 0 was present in 68% and 89% while half or more soft-tissue parenchyma was present in 7% and 0.2%, respectively. In a more recent study, the CT appearance of thymus was investigated in 597 otherwise healthy trauma patients recruited between 2015–2016 [14]. These authors reported a prevalence of complete fatty degeneration that was more similar to that in our cohort, 47% of patients aged 50–59 (*n* = 161) and 65% of patients aged 60–69 (*n* = 116). In accordance, they also reported a similar prevalence of residual thymic tissue (half or more soft-tissue parenchyma) in these two age categories, 22% and 12%, respectively.

As expected, male sex was a significant predictor of thymic involution. Previous studies based on CT assessments of thymus have reported that fatty degeneration of thymus is considerably more common in men than in women, both in young and elderly cohorts [13, 15]. Also, previous histological studies of thymus as well as studies of circulating TRECs have consistently shown a significant sex difference indicating that onset of thymic involution occurs 10–20 years later in women compared to men [27–29]. Intriguingly, it has been speculated that the consequence of delayed thymic involution in women may contribute to the difference in life expectancy seen between the sexes [29].

In addition to age and male sex, higher BMI levels and abdominal obesity were found to be strong predictors of complete fatty degeneration of thymus. The relationship between BMI and fat content in thymus has been reported in both young and elderly adults [13, 30]. Furthermore, the number of naïve T cells as well as TRECs have been shown to be lower in obese adults [31, 32]. Obesity is a multisystem disorder associated with low-grade chronic inflammation, compromised immune surveillance and accelerated aging [33–35]. Interestingly, experimental mouse models have shown that obesity induce acceleration of thymic aging, involving reduced production of naïve T cells, increased apoptosis of thymocytes and lower thymopoiesis [31]. In the present cohort, obesity-linked variables, such as diabetes, dyslipidemia (low HDL cholesterol and high triglyceride levels) and elevations in systemic inflammatory markers (CRP, IL-6 and IL-18), were more common among individuals with complete fatty degeneration of thymus. However, in multivariate regression models, all these variables lost their independent association with fatty degeneration of thymus, while BMI and abdominal obesity remained independent.

Former smoking, sedentary behavior and low intake of vitamins, minerals and fiber were all correlated with thymic scores in univariate analyses. Lifestyle factors are often referred to as determinants of healthy aging and there is experimental evidence that antioxidant-rich diet and exercise attenuate immunosenescence and thymic atrophy [3, 36]. Interestingly, Duggal et al. [37] reported higher levels of naïve T cells as well as T cell recent thymic emigrants in elderly master cyclists compared to elderly with a sedentary lifestyle. Not unexpectedly, considering the interrelationships between lifestyle factors, their significant correlations with thymus involution were lost in multiple regression models. However, there was one exception, namely the daily intake of fiber which remained independently associated with complete fatty degeneration of thymus. Of note, the positive effects of dietary fiber on microbiome and immune regulation are well established [38] and, in a large cohort of more than 5 600 US adults, higher fiber consumption was found to account for longer telomeres and less biological aging [39].

The proportions of naïve CD4^+^ T cells did not enter as independent determinants of thymic involution, which is in agreement with previous studies indicating that circulating pools of naïve CD4^+^ T cells in adults are not mainly dependent on thymic output, but rather well preserved due to peripheral proliferation [40–42]. On the other hand, diminished levels of naïve T_reg_ cells and particularly naïve CD8^+^ T cells remained independently associated with complete fatty degeneration of thymus. Interestingly, it has been reported previously that the compartment of naïve CD8^+^ T cell, in contrast to CD4^+^ T cells, shrinks markedly with age resulting in an almost complete loss in centenarians [41, 43]. Thus, representing the most profound age-related change of circulating T cells, the loss of naïve CD8^+^ T cells has been proposed to be a major hallmark of immunological aging [43]. Mechanisms underlying the loss of naïve CD8^+^ T cells are not clarified but may involve increased susceptibility to apoptosis and defective regeneration capacity [41, 44, 45]. Whether thymic function failure is involved in the loss of naïve CD8^+^ T cells has not been previously investigated. In the present study, TREC levels correlated with naïve T_reg_ cells and naïve CD8^+^ T cells, thus indicating that thymic output may contribute to the maintenance of these subsets.

The attenuation, size and morphology of thymus were measured in all participants with thymic scores ≥ 1, as previously described by Araki et al. [13]. Of note, CT attenuation values of the thymic gland were independent related to proportions of naïve CD8^+^ T cells. Obesity-related variables showed associations with both attenuation and thymic size in linear regression models, though in opposite directions. Thus, increased fat content may partly explain why thymic size appeared to be larger in individuals with lower thymic scores. However, data based on size measurements should be interpreted with great caution since neither size nor morphology of thymus have been considered reliable markers of thymic involution [13, 46].

Some other limitations of our study should be considered. A limited sample size may explain why certain variables, for example smoking status and diabetes, did not enter the regression models as independent determinants. Also, peripheral blood mononuclear cells for TREC measurements were available only from a small subgroup of participants. Self-reported data on dietary intake contain several potential sources of bias and caution should therefore be taken when interpreting them. The narrow age span in the study may be considered a strength but also a limitation since we were not able to thoroughly examine differences in associations across the lifespan. However, one important strength of the present study is the broad approach, combining the evaluation of lifestyle and biological factors in relation to measures of thymus imaging. Also, the importance of human studies should be pointed out since there are differences in thymic function across species, recently emphasized by Hellberg et al. [21] who showed that thymic output was maintained and even increased during human pregnancy.

In conclusion, CT assessments of thymus in a Swedish middle-aged population revealed that nearly two-thirds had a total fatty replacement of thymus. This phenomenon was strongly associated with male sex, obesity, low intake of dietary fiber as well as a depletion of naïve CD8^+^ T cells, the latter indicating that CT assessment of thymus can be a clinically relevant marker of immunological aging. Furthermore, our findings support the concept that obesity as well as low fiber intake contribute to immunological aging, thereby raising the possibility of prevention. This needs however to be verified in future prospective studies.

### Supplementary Information

**Additional file 1: Supplementary Table 1.** Summary of thymic measurements (mm) based on thymic score. **Supplementary Table 2.** Intra- and inter-reader agreement of thymic scores. **Supplementary Table 3.** Daily intake of micronutrients based on thymic scores. **Supplementary Table 4.** Correlations between fiber and micronutrients in all participants. **Supplementary Table 5.** Characteristics of male participants (n=530) based on thymic scores. **Supplementary Table 6.** Characteristics of female (n=518) participants based on thymic scores. **Supplementary Figure 1.** Gating strategy for the absolute counts of CD3+, CD4+ and CD8+ T cells in whole blood. A) Viable leukocytes and Trucount beads were identified in separate gates based on their locations in the plot, according to the manufacturer’s instruction. B) From the “Leukocyte” gate, granulocytes, monocytes and lymphocytes were gated based on their granularity and expression of CD45. C) From the “Lymphocytes” gate, CD3+ T cells were gated based on CD3 expression. D) From the “CD3+“ gate, CD4+ and CD8+ T cells were identified based on CD4 and CD8 expression, respectively. **Supplementary Figure 2.** Gating strategy for naïve CD4+ T cells, naive CD8+ T cells and naïve regulatory T cells (Treg cells). A) Viable lymphocytes were gated based on size and granularity. B) From the “Lymphocyte” gate, CD3+ T cells were identified based on CD3 expression. C) From the “CD3+“gate, CD4+ and CD8+ T cells were gated based on CD4 and CD8 expression, respectively. D) From the “CD4+” and “CD8+” gates, naive T cell subsets were gated defined as CD45RA+ CCR7+. E) From the “CD4+“ gate, naïve and memory T_reg_ cells were identified as CD25++CD45RA+ and CD25++CD45RA-, respectively. F) The naïve and memory T_reg_ cells identified in E) were backgated by displaying gate specific coloring on the CD45RA+ and CD45RA- populations in CD25 and side scatter (SSA) plots. T_reg_ cells were defined as CD25++ with relatively lower granularity compared to the rest of the T cells in the respective plots.

## Abbreviations

BMI: Body mass index
CRP: C-reactive protein
CT: Computed tomography
HbA1c: Glycated hemoglobin
HDL: High density lipoprotein
IL: Interleukin
LDL: Low density lipoprotein
SCAPIS: Swedish cardiopulmonary bio-image study
TREC: T cell receptor excision circles
T_reg_: Regulatory T cell
