# Supplementary material for: Complete fatty degeneration of thymus associates with male sex, obesity and loss of circulating naïve CD8+ T cells in a Swedish middle-aged population
Source: Immun Ageing. 2023 Aug 31;20:45. doi: 10.1186/s12979-023-00371-7 (PMC10470174; doi:10.1186/s12979-023-00371-7)
Supplement: Supplementary file 1 — Additional file 1: Supplementary Table 1. Summary of thymic measurements (mm) based on thymic score. Supplementary Table 2. Intra- and inter-reader agreement of thymic scores. Supplementary Table 3. Daily intake of micronutrients based on thymic scores. Supplementary Table 4. Correlations between fiber and micronutrients in all participants. Supplementary Table 5. Characteristics of male participants (n=530) based on thymic scores. Supplementary Table 6. Characteristics of female (n=518) participants based on thymic scores. Supplementary Figure 1. Gating strategy for the absolute counts of CD3+, CD4+ and CD8+ T cells in whole blood. A) Viable leukocytes and Trucount beads were identified in separate gates based on their locations in the plot, according to the manufacturer’s instruction. B) From the “Leukocyte” gate, granulocytes, monocytes and lymphocytes were gated based on their granularity and expression of CD45. C) From the “Lymphocytes” gate, CD3+ T cells were gated based on CD3 expression. D) From the “CD3+“ gate, CD4+ and CD8+ T cells were identified based on CD4 and CD8 expression, respectively. Supplementary Figure 2. Gating strategy for naïve CD4+ T cells, naive CD8+ T cells and naïve regulatory T cells (Treg cells). A) Viable lymphocytes were gated based on size and granularity. B) From the “Lymphocyte” gate, CD3+ T cells were identified based on CD3 expression. C) From the “CD3+“gate, CD4+ and CD8+ T cells were gated based on CD4 and CD8 expression, respectively. D) From the “CD4+” and “CD8+” gates, naive T cell subsets were gated defined as CD45RA+ CCR7+. E) From the “CD4+“ gate, naïve and memory Treg cells were identified as CD25++CD45RA+ and CD25++CD45RA-, respectively. F) The naïve and memory Treg cells identified in E) were backgated by displaying gate specific coloring on the CD45RA+ and CD45RA- populations in CD25 and side scatter (SSA) plots. Treg cells were defined as CD25++ with relatively lower granularity compared to the rest of the T cells i [file 12979_2023_371_MOESM1_ESM.docx]

**Supplementary material**

**Supplementary Table 1. Summary of thymic measurements (mm) based on thymic score.**

Subjects with any detectable thymic tissue are included in the analysis; Score 1, predominantly fatty thymus, Score 2, approximately one-half fatty and one-half soft tissue attenuation thymus, and Score 3, predominantly soft tissue attenuation thymus.

|  | **Score 1** (n=259) | **Score 2** (n=105) | **Score 3** (n=69) | **p**^a^ |
| --- | --- | --- | --- | --- |
| AP diameter | 29 (23-36) | 25 (20-30) | 24 (16-30) | <0.001 |
| Transverse diameter | 34 (28-41) | 30 (25-34) | 28 (24-34) | <0.001 |
| R, length | 22 (19-30) | 20 (16-26) | 18 (12-24) | <0.001 |
| R, thickness | 11 (8.2-13) | 9.1 (7.5-12) | 8.1 (6.2-10) | <0.001 |
| L, length | 34 (27-41) | 28 (22-35) | 27 (20-35) | <0.001 |
| L, thickness | 10 (8.1-13) | 9.3 (7.6-11) | 8.6 (7.0-10) | <0.001 |

AP, anteroposterior, R, right, L, left. Values are given as median (inter-quartile range).

^a^ The Kruskal-Wallis H test was used to compare characteristics between groups with different thymic scores.

**Supplementary Table 2. Intra- and inter-reader agreement of thymic scores.**

1. Intra-observer agreement of thymic score.

|  |  | | | | | | |
| --- | --- | --- | --- | --- | --- | --- | --- |
|  |  | | Observer 1 (First) | | | |  |
|  | **Thymic score** | |  | | | | Total |
|  |  |  | 0 | 1 | 2 | 3 |  |
| Observer 1 (Second) |  | 0 | 70 | 4 | 1 | 0 | 75 |
|  |  | 1 | 5 | 16 | 3 | 0 | 24 |
|  |  | 2 | 0 | 1 | 6 | 2 | 9 |
|  |  | 3 | 0 | 0 | 0 | 4 | 4 |
|  | Total | | 75 | 21 | 10 | 6 | 112 |

For intra-observer agreement between two sets of scores by one radiologist, the weighed Cohen kappa coefficient was 0.72 (95% CI: 0.59-0.84).

1. Inter-observer agreement of thymic score.

|  |  | | Observer 2 | | | |  |
| --- | --- | --- | --- | --- | --- | --- | --- |
|  | **Thymic score** | |  | | | | Total |
|  |  |  | 0 | 1 | 2 | 3 |  |
| Observer 1 |  | 0 | 66 | 8 | 1 | 0 | 75 |
|  |  | 1 | 3 | 19 | 2 | 0 | 24 |
|  |  | 2 | 0 | 2 | 7 | 0 | 9 |
|  |  | 3 | 0 | 0 | 3 | 1 | 4 |
|  | Total | | 75 | 69 | 29 | 13 | 1 |

For inter-observer agreement between two radiologists, the weighed Cohen kappa coefficient was 0.68 (95% CI: 0.54-0.79).

**Supplementary Table 3. Daily intake of micronutrients based on thymic scores.**

Daily intake of micronutrients is based on the food frequency questionnaire MiniMeal-Q. Four groups of thymic scores are presented; Score 0, complete fatty replacement, no identifiable soft tissue in the thymic bed, Score 1, predominantly fatty thymus, Score 2, approximately one-half fatty and one-half soft tissue attenuation thymus, and Score 3, predominantly soft tissue attenuation thymus.

|  | **0 (n=615)** | **1 (n=258)** | **2 (n=103)** | **3 (n=67)** | p^a^ |
| --- | --- | --- | --- | --- | --- |
| **Calculated nutrients** | | | | | |
| Vitamin A | 0.63 (0.43-0.86) | 0.67 (0.48-0.90) | 0.73 (0.49-0.87) | 0.68 (0.46-0.92) | 0.106 |
| Retinol | 0.29 (0.21-0.41) | 0.28 (0.21-0.36) | 0.27 (0.22-0.40) | 0.27 (0.19-0.40) | 0.349 |
| β-carotene (μg) | 2837 (1669-4829) | 3502 (1959-5505) | 3691 (2227-6260) | 3768 (1988-6625) | <0.001 |
| Vitamin D | 5.6 (3.9-7.7) | 5.3 (3.7-7.7) | 5.7 (4.3-7.6) | 4.9 (4.0-6.5) | 0.356 |
| Vitamin E (mg) | 8.2 (6.1-11) | 8.7 (6.6-12) | 9.4 (7.2-12) | 9.1 (6.1-11) | 0.006 |
| Vitamin K (μg) | 24 (17-35) | 27 (20-38) | 28 (20-42) | 28 (19-39) | <0.001 |
| Thiamine (mg) | 1.1 (0.8-1.4) | 1.2 (0.9-1.5) | 1.2 (0.9-1.6) | 1.2 (0.9-1.6) | 0.003 |
| Riboflavin | 1.44 (1.05-1.89) | 1.53 (1.17-1.92) | 1.53 (1.09-1.95) | 1.50 (1.02-1.91) | 0.232 |
| Vitamin C | 72 (47-101) | 80 (54-111) | 73 (49-110) | 88 (57-111) | 0.042 |
| Niacin | 16 (13-20) | 16 (13-20) | 16 (13-19) | 16 (13-20) | 0.893 |
| Vitamin B6 | 1.54 (1.20-1.92) | 1.62 (1.33-2.07) | 1.72 (1.36-2.06) | 1.53 (1.16-2.15) | 0.010 |
| Vitamin B12 | 3.96 (2.87-5.19) | 3.88 (2.89-5.26) | 4.35 (2.99-5.30) | 3.76 (2.93-4.68) | 0.456 |
| Phosphorus | 1228 (945-1563) | 1267 (998-1624) | 1349 (1100-1547) | 1172 (1025-1615) | 0.191 |
| Folate (μg) | 276 (213-356) | 307 (241-382) | 319 (252-377) | 307 (234-379) | <0.001 |
| Iron (mg) | 8.9 (6.7-12) | 10 (8.0-13) | 10 (7.4-14) | 9.7 (7.6-12) | 0.004 |
| Calcium | 826 (595-1101) | 847 (636-1132) | 892 (652-1045) | 788 (636-1071) | 0.646 |
| Potassium | 2812 (2287-3397) | 2915 (2469-3612) | 3072 (2426-3542) | 2744 (2175-7740) | 0.045 |
| Magnesium mg) | 303 (245-384) | 332 (270-418) | 361 (273-420) | 329 (249-422) | <0.001 |
| Sodium | 2078 (1641-2620) | 2095 (1652-2593) | 2133 (1731-2579) | 1975 (1624-2452) | 0.531 |
| Selenium | 40 (29-54) | 41 (30-65) | 48 (30-69) | 41 (30-55) | 0.013 |
| Zinc | 8.6 (6.9-11) | 8.8 (7.0-11) | 9.4 (7.4-11) | 8.3 (6.8-11) | 0.339 |

**Supplementary Table 4. Correlations between fiber and micronutrients in all participants.**

Daily intake of micronutrients is based on the food frequency questionnaire MiniMeal-Q. The micronutrients presented in Table 2 are included in the analysis.

|  | Fiber | β-carotene | Vitamin E | Vitamin K | Thiamine | Folate | Iron | Magnesium |
| --- | --- | --- | --- | --- | --- | --- | --- | --- |
| Fiber | - | - | - | - | - | - | - | - |
| β-carotene | 0.550^**^ | - | - | - | - | - | - | - |
| Vitamin E | 0.746^**^ | 0.528^**^ | - | - | - | - | - | - |
| Vitamin K | 0.602^**^ | 0.672^**^ | 0.752^**^ | - | - | - | - | - |
| Thiamine | 0.747^**^ | 0.399^**^ | 0.682^**^ | 0.481^**^ | - | - | - | - |
| Folate | 0.801^**^ | 0.575^**^ | 0.777^**^ | 0.720^**^ | 0.799^**^ | - | - | - |
| Iron | 0.759^**^ | 0.415^**^ | 0.729^**^ | 0.571^**^ | 0.821^**^ | 0.755^**^ | - | - |
| Magnesium | 0.809^**^ | 0.454^**^ | 0.787^**^ | 0.590^**^ | 0.830^**^ | 0.835^**^ | 0.868^**^ | - |

^**^ Spearman correlation coefficient, p< 0.001

**Supplementary Table 5.** Characteristics of male participants (n=530) based on thymic scores.

Four groups of thymic scores are presented; Score 0, complete fatty replacement, no identifiable soft tissue in the thymic bed, Score 1, predominantly fatty thymus, Score 2, approximately one-half fatty and one-half soft tissue attenuation thymus, and Score 3, predominantly soft tissue attenuation thymus.

**Readers should be mindful of small sample sizes in groups with Score 2 and 3 affecting statistical interpretation***.*

|  | **Thymic scores** | | | | |
| --- | --- | --- | --- | --- | --- |
|  | 0 (n=391) | 1 (n=103) | 2 (n=26) | 3 (n=10) | p^a^ |
| *Demographic and clinical characteristics* | | | | |  |
| Age, years | 58 (55-62) | 55 (52-60) | 57 (53-59) | 56 (53-62) | <0.001 |
| BMI | 27 (25-30) | 26 (24-28) | 25 (23-26) | 24 (23-26) | <0.001 |
| Abdominal obesity | 350 (90) | 73 (71) | 15 (58) | 6 (60) | <0.001 |
| Smoking, current  former | 32 (8.2) | 3 (2.9) | 2 (7.7) | 1 (10) | NS |
|  | 120 (31) | 21 (20) | 4 (15) | 1 (10) | 0.045 |
| Anti-hypertensive medication | 96 (25) | 15 (15) | 1 (3.8) | 1 (10) | NS |
| Hypertension, systolic^b^  diastolic^c^ | 141 (36) | 29 (28) | 7 (2**7**) | 3 (30) | NS |
|  | 110 (28) | 18 (17) | 6 (23) | 1 (10) | NS |
| Anti-diabetic medication | 28 (7.2) | 1 (1.0) | 1 (3.8) | 1 (10) | NS |
| Diabetes^d^ | 23 (5.9) | 1 (1.0) | 1 (3.8) | 1 (10) | NS |
| *Clinical chemistry* | | | | | |
| Total cholesterol, mmol/L | 5.3 (4.5-6.0) | 5.2 (4.6-5.8) | 5.2 (4.6-6.0) | 5.1 (4.3-5.6) | NS |
| LDL cholesterol, mmol/L | 3.2 (2.6-4.0) | 3.2 (2.8-3.8) | 3.3 (2.6-3.7) | 2.6 (2.2-3.3) | NS |
| HDL cholesterol, mmol/L | 1.4 (1.1-1.6) | 1.4 (1.2-1.7) | 1.5 (1.2-1.7) | 2.1 (1.6-2.3) | <0.001 |
| Triglycerides, mmol/L | 1.2 (0.9-1.8) | 1.1 (0.8-1.5) | 1.1 (0.8-1.3) | 0.6 (0.4-1.1) | <0.001 |
| CRP, mg/L | 1.0 (0.5-2.0) | 0.9 (0.4-1.6) | 0.6 (0.3-1.0) | 0.8 (0.4-1.7) | 0.013 |
| IL-6, pg/mL | 1.13 (0.78-1.50) | 1.00 (0.67-1.36) | 0.89 (0.58-1.24) | 1.15 (1.00-1.77) | 0.043 |
| IL-18, pg/mL | 390 (312-507) | 374 (299-469) | 423 (284-586) | 328 (249-377) | NS |
| *White blood cell differential counts* | | | | |  |
| Leukocytes, 10^3^/μL | 6.0 (5.1-7.1) | 5.6 (4.7-6.2) | 5.4 (4.7-6.3) | 5.2 (4.4-6.1) | 0.003 |
| Granulocytes, 10^3^/μL | 3.5 (2.9-4.4) | 3.1 (2.7-3.6) | 3.1 (2.6-3.6) | 2.6 (2.1-3.7) | NS |
| Lymphocytes, 10^3^/μL | 1.8 (1.4-2.1) | 1.8 (1.4-2.1) | 1.8 (1.4-2.3) | 1.7 (1.4-2.0) | NS |
| *Physical activity, % of daily wear time* | | | | | |
| Sedentary | 58 (51-64) | 57 (52-63) | 57 (51-60) | 51 (42-58) | NS |
| Low intensity | 36 (31-43) | 36 (31-43) | 36 (32-44) | 42 (35-53) | NS |
| Moderate-vigorous | 6.0 (4.0-7.0) | 6.0 (4.0-8.0) | 6.5 (4.5-8.3) | 7.0 (4.8-8.3) | NS |
| *Calculated nutrients* | | | | | |
| Energy (kcal) | 1647 (1300-2087) | 1712 (1385-2135) | 1782 (1480-2022) | 1713 (1268-2912) | NS |
| Protein (g) | 68 (54-83) | 68 (55-88) | 71 (59-82) | 59 (47-101) | NS |
| Carbohydrates (g) | 172 (133-227) | 186 (145-239) | 177 (154-208) | 226 (140-321) | NS |
| Total fat (g) | 65 (48-83) | 64 (50-83) | 74 (54-84) | 59 (35-127) | NS |
| Fiber (g) | 16 (11-22) | 19 (13-25) | 20 (14-26) | 21 (12-30) | 0.006 |
| β-carotene (μg) | 2534 (1591-4142) | 3072 (1840-4908) | 3510 (1700-6690) | 3117 (1002-5516) | NS |
| Vitamin E (mg) | 8.1 (6.1-11) | 8.7 (6.5-11) | 10 (7.8-13) | 8.7 (6.0-11) | NS |
| Vitamin K (μg) | 23 (16-32) | 24 (18-36) | 29 (18-46) | 27 (12-37) | NS |
| Thiamine (mg) | 1.1 (0.9-1.4) | 1.3 (1.0-1.6) | 1.3 (0.9-1.9) | 1.3 (0.9-1.8) | 0.046 |
| Folate (μg) | 275 (209-357) | 290 (219-392) | 316 (267-381) | 314 (226-391) | NS |
| Iron (mg) | 9.2 (6.7-12) | 11 (8.2-13) | 12 (8.2-14) | 12 (8.1-14) | 0.007 |
| Magnesium mg) | 307 (248-384) | 333 (271-426) | 378 (291-423) | 364 (232-4479) | 0.031 |
| *Flow cytometric data on CD3^+^ T cells and T cell subsets* | | | | | |
| CD3^+^ T cells/μL | 1291 (1059-1598) | 1286 (1043-1611) | 1350 (1198-1664) | 1270 (1122-1667) | NS |
| CD4^+^ T cells/μL | 834 (660-1015) | 770 (616-1019) | 781 (634-1164) | 776 (680-937) | NS |
| CD8^+^ T cells/μL | 417 (285-593) | 446 (304-559) | 467 (325-653) | 488 (254-726) | NS |
| CD4^+^ T cells, % of CD3^+^ | 48 (42-54) | 47 (41-53) | 48 (45-52) | 46 (36-54) | NS |
| CD8^+^ T cells, % of CD3^+^ | 24 (18-31) | 25 (19-31) | 28 (22-34) | 29 (20-40) | NS |
| T_reg_ cells, % of CD4^+^ cells | 3.8 (3.2-4.7) | 4.0 (3.3-4.9) | 4.1 (3.5-5.2) | 4.4 (3.2-5.2) | NS |
| Naive CD4^+^ T cells, % of CD4^+^ | 38 (26-49) | 44 (34-54) | 46 (35-54) | 46 (33-59) | <0.001 |
| Naïve CD8^+^ T cells, % of CD8^+^ | 12 (6.9-20) | 18 (9.7-28) | 18 (12-34) | 16 (14-39) | <0.001 |
| Naïve T_reg_ cells, % of CD4^+^ cells | 1.4 (1.0-1.8) | 1.8 (1.3-2.2) | 1.8 (1.4-2.2) | 2.0 (1.5-2.7) | <0.001 |

Continuous data are expressed as median (interquartile range) and dichotomous data as n (%). ^a^The Kruskal-Wallis H test was used to compare characteristics between groups with different thymic scores. ^b^Systolic hypertension, defined as > 140 mm Hg. ^c^Diastolic hypertension, defined as > 90 mm Hg. ^d^Diabetes, defined as HbA1c > 48 mmol/mol.

**Supplementary Table 6.** Characteristics of female (n=518) participants based on thymic scores.

Four groups of thymic scores are presented; Score 0, complete fatty replacement, no identifiable soft tissue in the thymic bed, Score 1, predominantly fatty thymus, Score 2, approximately one-half fatty and one-half soft tissue attenuation thymus, and Score 3, predominantly soft tissue attenuation thymus.

|  | **Thymic scores** | | | | |
| --- | --- | --- | --- | --- | --- |
|  | 0 (n=224) | 1 (n=156) | 2 (n=79) | 3 (n=59) | p^a^ |
| *Demographic and clinical characteristics* | | | | |  |
| Age, years | 59 (54-62) | 56 (53-60) | 54 (51-58) | 54 (52-58) | <0.001 |
| BMI | 27 (24-31) | 25 (23-28) | 24 (22-27) | 24 (21-26) | <0.001 |
| Abdominal obesity | 138 (62) | 71 (46) | 25 (32) | 10 (17) | <0.001 |
| Smoking, current  former | 20 (8.9) | 9 (5.8) | 4 (5.1) | 3 (5.1) | NS |
|  | 84 (38) | 50 (32) | 22 (28) | 10 (17) | 0.020 |
| Anti-hypertensive medication | 40 (18) | 23 (15) | 2 (2.5) | 9 (15) | NS |
| Hypertension, systolic^b^  diastolic^c^ | 71 (32) | 41 (26) | 13 (16) | 13 (22) | NS (0.051) |
|  | 66 (29) | 37 (24) | 11 (14) | 12 (20) | 0.038 |
| Anti-diabetic medication | 12 (5.4) | 3 (1.9) | 2 (2.5) | 1 (1.7) | NS |
| Diabetes^d^ | 11 (4.9) | 1 (0.6) | 2 (2.5) | 1 (1.7) | NS |
| *Clinical chemistry* | | | | | |
| Total cholesterol, mmol/L | 5.7 (4.8-6.6) | 5.6 (5.0-6.2) | 5.6 (4.9-6.3) | 5.3 (4.9-5.9) | NS |
| LDL cholesterol, mmol/L | 3.3 (2.6-4.1) | 3.2 (2.7-3.8) | 3.2 (2.5-3.9) | 2.9 (2.3-3.3) | 0.011 |
| HDL cholesterol, mmol/L | 1.7 (1.4-2.1) | 1.8 (1.6-2.2) | 1.9 (1.6-2.2) | 2.0 (1.8-2.4) | <0.001 |
| Triglycerides, mmol/L | 1.1 (0.8-1.5) | 0.9 (0.7-1.2) | 0.9 (0.7-1.2) | 0.8 (0.6-1.0) | <0.001 |
| CRP, mg/L | 1.3 (0.6-3.0) | 0.8 (0.4-1.8) | 0.7 (0.4-1.4) | 0.7 (0.3-1.4) | <0.001 |
| IL-6, pg/mL | 1.24 (0.84-1.69) | 1.02 (0.68-1.47) | 1.00 (0.72-1.43) | 0.96 (0.76-1.30) | 0.014 |
| IL-18, pg/mL | 319 (247-415) | 294 (237-365) | 299 (231-399) | 269 (222-363) | 0.034 |
| *White blood cell differential counts* | | | | |  |
| Leukocytes, 10^3^/μL | 5.8 (5.0-6.8) | 5.5 (4.7-6.6) | 5.3 (4.3-6.5) | 5.5 (4.4-6.4) | 0.049 |
| Granulocytes, 10^3^/μL | 3.3 (2.7-4.0) | 3.0 (2.4-3.9) | 2.9 (2.2-3.8) | 2.9 (2.3-3.8) | 0.001 |
| Lymphocytes, 10^3^/μL | 1.8 (1.5-2.1) | 1.7 (1.5-2.2) | 1.9 (1.5-2.2) | 1.7 (1.5-2.1) | NS |
| *Physical activity, % of daily wear time* | | | | | |
| Sedentary | 53 (46-60) | 53 (45-59) | 51 (44-58) | 52 (46-57) | NS |
| Low intensity | 41 (35-48) | 41 (36-47) | 42 (36-47) | 41 (36-46) | NS |
| Moderate-vigorous | 5.0 (4.0-7.0) | 6.0 (4.0-8.0) | 7.0 (5.0-8.0) | 7.0 (5.0-9.0) | < 0.001 |
| *Calculated nutrients* | | | | | |
| Energy (kcal) | 1416 (1428-1841) | 1505(1232-1871) | 1630 (1241-1843) | 1430 (1161-1895) | NS |
| Protein (g) | 58 (46-73) | 61 (49-76) | 65 (51-77) | 58 (50-75) | NS |
| Carbohydrates (g) | 147 (116-204) | 160 (128-199) | 166 (134-202) | 152 (120-213) | NS |
| Total fat (g) | 57 (45-77) | 62 (46-80) | 64 (51-79) | 54 (45-77) | NS |
| Fiber (g) | 17 (12-23) | 19 (14-25) | 19 (14-25) | 19 (13-25) | 0.022 |
| β-carotene (μg) | 3727 (1889-6077) | 3915 (2192-6002) | 3719 (2289-6150) | 3840 (2246-6726) | NS |
| Vitamin E (mg) | 8.3 (6.1-11) | 8.8 (6.7-12) | 9.3 (7.0-12) | 9.1 (6.0-11) | NS |
| Vitamin K (μg) | 27 (18-41) | 30 (22-41) | 28 (20-41) | 28 (20-40) | NS |
| Thiamine (mg) | 1.1 (0.8-1.4) | 1.2 (0.9-1.5) | 1.2 (0.9-1.6) | 1.2 (0.9-1.6) | 0.003 |
| Folate (μg) | 278 (220-356) | 315 (251-381) | 319 (250-374) | 303 (234-380) | 0.012 |
| Iron (mg) | 8.7 (6.8-12) | 9.7 (7.6-12) | 9.8 (7.3-13) | 9.2 (7.4-12) | NS |
| Magnesium mg) | 296 (242-381) | 331 (269-417) | 352 (271-424) | 320 (250-429) | 0.013 |
| *Flow cytometric data on CD3^+^ T cells and T cell subsets* | | | | | |
| CD3^+^ T cells/μL | 1364 (1094-1631) | 1354 (1115-1688) | 1474 (1192-1715) | 1306 (1080-1666) | NS |
| CD4^+^ T cells/μL | 892 (736-1109) | 901 (731-1154) | 925 (769-1169) | 903 (669-1093) | NS |
| CD8^+^ T cells/μL | 391 (294-566) | 436 (322-583) | 436 (336-589) | 407 (342-524) | NS |
| CD4^+^ T cells, % of CD3^+^ | 51 (45-58) | 52 (45-57) | 53 (48-57) | 49 (44-56) | NS |
| CD8^+^ T cells, % of CD3^+^ | 23 (18-29) | 25 (20-30) | 24 (19-30) | 25 (19-33) | NS |
| T_reg_ cells, % of CD4^+^ cells | 3.8 (3.1-4.5) | 4.0 (3.3-4.6) | 4.2 (3.3-4.8) | 3.8 (3.3-4.6) | NS |
| Naive CD4^+^ T cells, % of CD4^+^ | 42 (31-52) | 46 (36-54) | 50 (40-60) | 46 (37-56) | <0.001 |
| Naïve CD8^+^ T cells, % of CD8^+^ | 14 (7.4-23) | 20 (14-33) | 27 (16-38) | 34 (16-44) | <0.001 |
| Naïve T_reg_ cells, % of CD4^+^ cells | 1.4 (1.0-1.8) | 1.5 (1.1-2.1 | 1.6 (1.3-2.2) | 1.8 (1.4-2.2) | <0.001 |

Continuous data are expressed as median (interquartile range) and dichotomous data as n (%). ^a^The Kruskal-Wallis H test was used to compare characteristics between groups with different thymic scores. ^b^Systolic hypertension, defined as > 140 mm Hg. ^c^Diastolic hypertension, defined as > 90 mm Hg. ^d^Diabetes, defined as HbA1c > 48 mmol/mol.

**
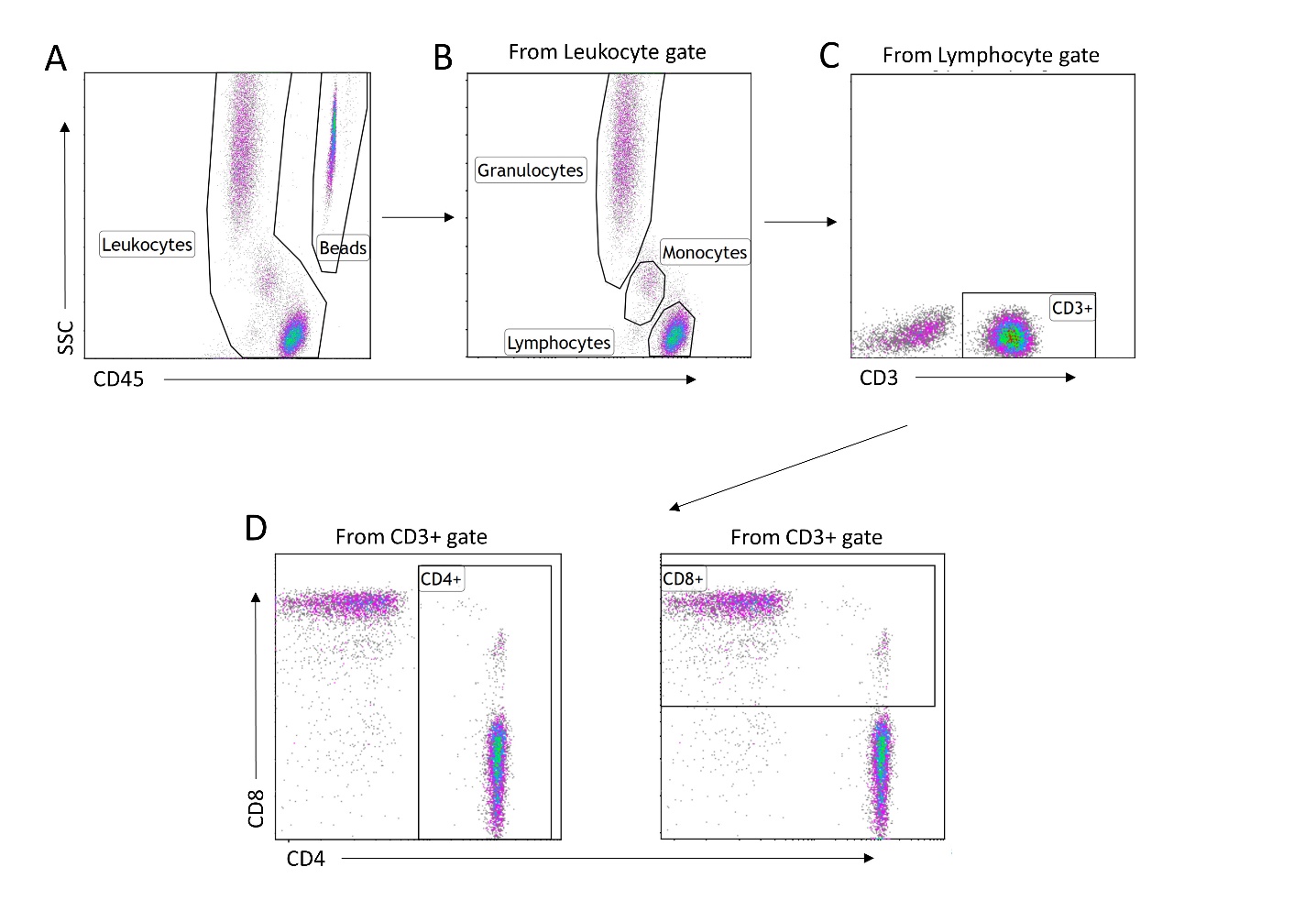
**

**Supplementary Figure 1.** Gating strategy for the absolute counts of CD3^+^, CD4^+^ and CD8^+^ T cells in whole blood. A) Viable leukocytes and Trucount beads were identified in separate gates based on their locations in the plot, according to the manufacturer’s instruction. B) From the “Leukocyte” gate, granulocytes, monocytes and lymphocytes were gated based on their granularity and expression of CD45. C) From the “Lymphocytes” gate, CD3^+^ T cells were gated based on CD3 expression. D) From the “CD3^+^“ gate, CD4^+^ and CD8^+^ T cells were identified based on CD4 and CD8 expression, respectively.

**
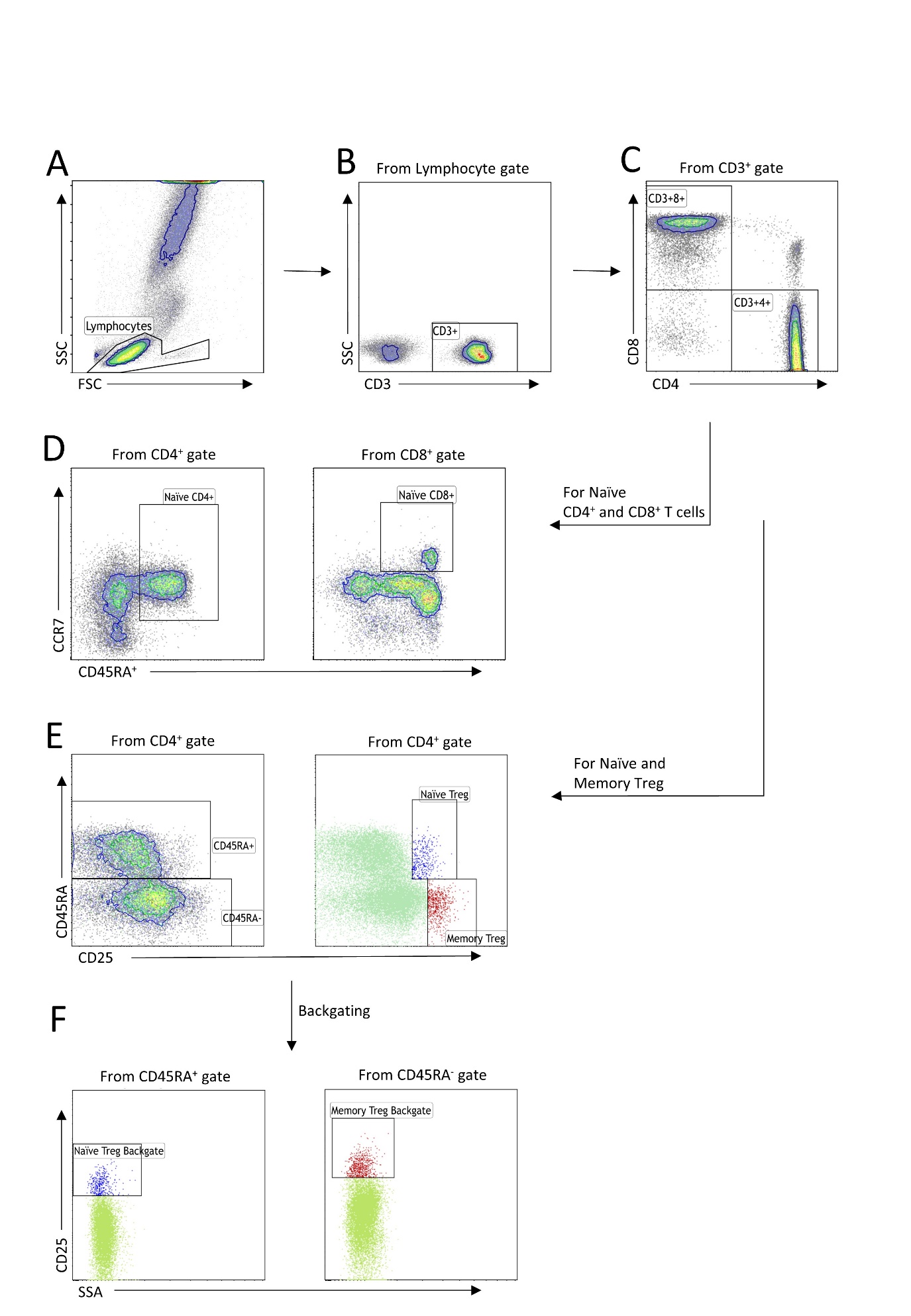
**

**Supplementary Figure 2**. Gating strategy for naïve CD4+ T cells, naive CD8+ T cells and naïve regulatory T cells (T_reg_ cells). A) Viable lymphocytes were gated based on size and granularity. B) From the “Lymphocyte” gate, CD3^+^ T cells were identified based on CD3 expression. C) From the “CD3^+^“gate, CD4^+^ and CD8^+^ T cells were gated based on CD4 and CD8 expression, respectively. D) From the “CD4^+^” and “CD8^+^” gates, naive T cell subsets were gated defined as CD45RA^+^ CCR7^+^. E) From the “CD4^+^“ gate, naïve and memory T_reg_ cells were identified as CD25^++^CD45RA^+^ and CD25^++^CD45RA^-^, respectively. F) The naïve and memory T_reg_ cells identified in E) were backgated by displaying gate specific coloring on the CD45RA^+^ and CD45RA^-^ populations in CD25 and side scatter (SSA) plots. T_reg_ cells were defined as CD25++ with relatively lower granularity compared to the rest of the T cells in the respective plots.
